# Androgen Receptor Signaling in Salivary Gland Cancer

**DOI:** 10.3390/cancers9020017

**Published:** 2017-02-08

**Authors:** Martin G. Dalin, Philip A. Watson, Alan L. Ho, Luc G. T. Morris

**Affiliations:** 1 Human Oncology and Pathogenesis Program, Memorial Sloan Kettering Cancer Center, New York, NY 10065, USA; watsonp@mskcc.org; 2 Department of Pediatrics, Institution for Clinical Sciences, University of Gothenburg, Gothenburg SE-416 86, Sweden; 3 Head and Neck Medical Oncology Service, Department of Medicine, Memorial Sloan Kettering Cancer Center, New York, NY 10065, USA; hoa@mskcc.org; 4 Head and Neck Service, Department of Surgery, Memorial Sloan Kettering Cancer Center, New York, NY 10065, USA

**Keywords:** salivary gland cancer, androgen receptor, salivary duct carcinoma, androgen-deprivation therapy (ADT)

## Abstract

Salivary gland cancers comprise a small subset of human malignancies, and are classified into multiple subtypes that exhibit diverse histology, molecular biology and clinical presentation. Local disease is potentially curable with surgery, which may be combined with adjuvant radiotherapy. However, metastatic or unresectable tumors rarely respond to chemotherapy and carry a poorer prognosis. Recent molecular studies have shown evidence of androgen receptor signaling in several types of salivary gland cancer, mainly salivary duct carcinoma. Successful treatment with anti-androgen therapy in other androgen receptor-positive malignancies such as prostate and breast cancer has inspired researchers to investigate this treatment in salivary gland cancer as well. In this review, we describe the prevalence, biology, and therapeutic implications of androgen receptor signaling in salivary gland cancer.

## 1. Introduction

Salivary gland cancers (SGCs) are a group of uncommon, heterogeneous tumors that account for 0.3% of all malignancies and 6% of head and neck cancers in the United States [1]. The majority of SGCs are found in the parotid gland (59%–81% of cases), but they also arise in the submandibular gland (6%–21%), or in minor salivary glands (7%–22%) that populate the upper aerodigestive tract [2,3,4]. The World Health Organization classifies 24 subtypes of SGC, which show significant variation in histological and clinical features [1]. SGC is generally treated with surgery and, in selected cases, adjuvant radiotherapy (RT) [5]. Systemic therapy has not been adequately tested in many SGC subtypes, and cytotoxic chemotherapy has shown a limited effect in SGCs in general. As a consequence, the prognosis of recurrent or metastatic SGC can be poor [2,6,7]. However, recent studies have investigated the molecular landscape of several types of SGCs, such as adenoid cystic carcinoma (ACC), mucoepidermoid carcinoma (MEC), polymorphous low grade adenocarcinoma (PLGA), secretory carcinoma and salivary duct carcinoma (SDC), and uncovered molecular targets of interest in selected patients [8,9,10,11,12,13].

The androgen receptor (AR) is a nuclear steroid hormone receptor that is physiologically expressed at low levels in many human tissues [14]. Its main ligands are testosterone and 5α-dihydrotestosterone (DHT). AR regulates the transcription of multiple effector genes through direct DNA binding or interaction with other transcription factors, leading to increased cell growth, differentiation, and survival [15]. Overactive AR signaling is an important oncogenic driver in several tumor types, including prostate cancer and a subset of breast cancers [16,17]. Androgen-deprivation therapy (ADT) has been used in patients with prostate cancer since the 1940s [18], and has more recently gained interest in a growing number of malignancies [17,19,20,21]. ADT may be achieved by direct inhibition of AR (known as anti-androgen therapy), or by downregulating the gonadotropin-releasing hormone (GnRH) receptor signaling output, which leads to reduced serum testosterone levels (known as chemical castration). These two methods are often combined to achieve what has been termed maximum or complete androgen blockade [22].

## 2. AR Expression in SGC

Nuclear AR expression based on immunohistochemistry (IHC) is the most widely used marker of active AR signaling, and correlates with the response to ADT in prostate cancer [23]. The prevalence of AR expression varies substantially between different subtypes of SGC (see Table 1 for a summary of published IHC data). AR overexpression is most frequently associated with salivary duct carcinomas (SDC), the majority of which are positive for AR. Several studies have shown AR immunoreactivity in 64%–77% of cases [8,24,25,26,27,28,29,30], whereas a recent large report detected AR expression in as many as 98% of SDCs [31]. In that study, several tumors initially diagnosed as AR-negative SDCs were reclassified as other tumor entities after a second evaluation by salivary pathologists. Also, for tumors with conventional SDC morphology and a negative first AR IHC, the staining was repeated and showed AR expression the second time in several cases. This may suggest that the prevalence of AR-positive SDC was previously underestimated due to technical issues or diagnostic difficulties.

Our group recently identified AR positivity by IHC in 75% of SDCs, and RNA sequencing confirmed extremely low but detectable levels of AR mRNA in AR IHC–negative cases, all of which had typical SDC morphology at the time of pathologic re-evaluation [8]. Interestingly, three of four AR IHC-negative cases showed AR signaling activity at levels equivalent to AR IHC-positive cases, as measured by expression of AR-regulated genes. Both AR-negative and AR-positive SDCs showed global gene expression patterns highly similar to AR-positive (also termed molecular apocrine) breast cancers. This raises the possibility that some SDCs with low levels of AR may have acquired alternative mechanisms to activate AR signaling pathways. Furthermore, the remarkable biological similarity between the two cancer types may suggest that treatment results in patients with molecular apocrine breast cancer could be of interest for the design of clinical trials in SDC.

The prognostic relevance of AR expression in SDC is difficult to assess, due to the rarity of the disease and the low number of AR-negative cases. Some investigators have identified a trend suggestive of better disease-free survival in AR-positive compared to AR-negative SDC patients [26,29], but this association has not been identified by other groups [8,24,25]. Similarly, one study detected a higher prevalence of AR expression in men than in women with SDC [30], a finding that has not been replicated in other reports [8,26].

In other subtypes of SGC, nuclear AR expression is found at lower rates. Adenocarcinoma, not otherwise specified (AC NOS) and acinic cell carcinoma (AcCC) are AR-positive in 26% and 15% of the cases, respectively [28,32,33,34,35]. On the other hand, only a small subset of MEC and ACC have detectable expression of AR [27,28,32,33,34,36,37], and some of these cases show weak AR expression (5%–15% stained nuclei) which may not be relevant for the biology of the tumors [32]. Among the rare types of SGC, AR expression has been reported in PLGA and basal cell adenocarcinoma (BCAC) [28,32], whereas all published cases of myoepithelial carcinoma (MECA) have been AR-negative [28,33]. Five cases of AR-positive epithelial-myoepithelial carcinoma (EMC) were reported and suggested to represent a specific variant of the disease, denoted apocrine EMC [38]. However, one study of six unselected EMCs did not detect AR [28], and the prevalence of AR expression in EMC is unknown. Given the challenging nature of salivary gland pathology, it is possible that some of these AR-positive entities in fact represent SDC.

A subset of SGCs result from the malignant transformation of a pre-existing pleomorphic adenoma (PA). PA is the most prevalent salivary gland tumor, and is typically benign and non-metastatic. Around 6% of PAs develop into different types of carcinoma, denoted carcinoma ex-PA [39]. Whereas PAs are AR-positive in 30% of the cases, 90% of carcinoma ex-PAs express AR. This difference may suggest that AR expression is a risk factor for the malignant transformation of PAs. Alternatively, overexpression of AR may act as an oncogenic event in some carcinomas ex-PA [40].

## 3. Expression of AR Splice Variants

**Figure 1:**
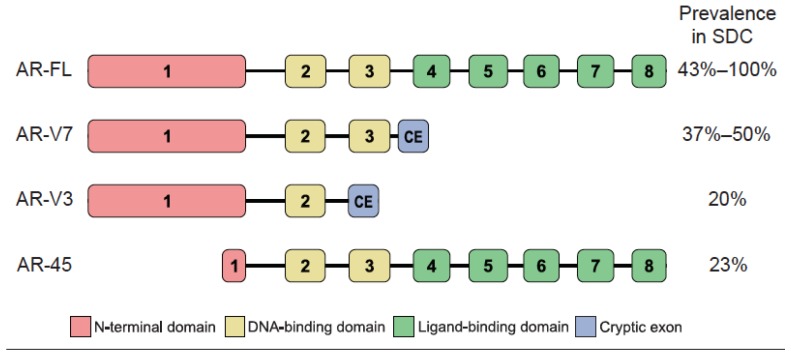
Reported prevalence of AR splice variant expression in SDC. References: For AR-FL, [8,24,25,26,27,28,29,30,31,33,34,41,42,43,44,45]; for AR-V7, [8,26]; for AR-V3 and AR-45, [26].

The full-length AR (AR-FL) gene consists of eight exons, of which exons 4–8 encode the ligand-binding domain. Expression of alternative AR isoforms lacking the ligand-binding domain (which normally serves as a binding site for anti-androgens, such as enzalutamide) is associated with ADT resistance in prostate cancer [46,47,48,49,50]. AR-V7, a constitutively active AR splice variant that includes only exons 1–3 and a cryptic exon 3, is detected in 37%–50% of SDCs (Figure 1) [8,26]. On average, AR-V7 is expressed at around 5% of AR-FL RNA levels [8], which is similar to the AR-V7/AR-FL ratio seen in prostate cancer [16]. Another AR isoform, AR-V3, including only exons 1, 2 and a cryptic exon 2, is also found in SDC but at lower rates and only in male patients [26]. AR-45, which lacks the majority of exon 1, including the N-terminal domain that mediates ligand-independent transactivation of AR [51], is detected in a minority of SDCs [26]. However, the association between the alternative AR isoforms and response to ADT in SDC, and the prevalence of AR-V7, AR-V3, and AR-45 in other types of SGC, remains unknown.

## 4. Genetic Alterations Affecting AR Signaling

An extra copy of chromosome X, which includes the AR gene, is found in almost 40% of SDCs. This may contribute to overexpression of AR, although some of the tumors with an extra chromosome X are negative for AR in IHC [26]. Unlike in prostate cancer, focal amplification or protein-altering somatic mutations of AR have not been found in SDC or ACC [8,9,26].

Forkhead box protein A1 (FOXA1) is a transcription factor that mediates the transcription of AR target genes by facilitating the AR/chromatin interaction [52]. *FOXA1* mutations may potentially be associated with ADT resistance in prostate cancer, although this is being actively investigated [53]. In a recent exome sequencing study reported by our group, we identified alteration (either somatic mutations in the DNA-binding domain or high-level amplification) of *FOXA1* in four of 12 AR-positive SDCs. Conversely, no *FOXA1* alterations were found in four AR-negative SDCs [8].

Fatty acid synthase (FASN) is an enzyme that controls fatty acid synthesis and has been shown to promote the growth of prostate cancer as a result of AR signaling. Experimental studies suggest that *FASN* overexpression can mediate resistance to ADT in prostate cancer, although no clinical data are yet available [54]. In our exome study of SDC, alterations (missense mutations, a frameshift insertion, and high-level amplification) of *FASN* were found in four of 12 AR-positive but not in AR-negative tumors [8].

In ACC, which rarely expresses AR, no significant genetic alterations affecting AR signaling have been detected [9]. In other subtypes of SGC, the prevalence of AR-related genetic alterations is unknown.

## 5. Anti-Androgen Therapy in Patients with SGC

Several ADT drugs have been developed and tested clinically, mainly in patients with prostate cancer. Abiraterone is a CYP17A1 inhibitor which reduces circulating levels of androgen by ultimately blocking the conversion of pregnenolone to DHT. Bicalutamide and flutamide are competitive inhibitors of the AR ligand-binding domain, as is enzalutamide, which was developed more recently and has greater AR affinity compared to the earlier anti-androgens, and may inhibit AR activity via a variety of different mechanisms [55]. Triptorelin and goserelin are GnRH agonists which eventually cause downregulation of luteinizing hormone (LH) and thereby reduced serum testosterone levels [22].

Inspired by results from other cancers [17,56] and functional studies showing AR-dependency in cultured SGC cells [26,57], a number of patients with AR-positive SGC have been treated with different ADT regimens (see Table 2 for a summary of reported cases). In a retrospective analysis of 17 patients with recurrent or metastatic AR-positive SGC, of which the majority had SDC or AC NOS, the overall response rate was 65%. Treatment was generally well tolerated in these patients, both men and women. However, relapse was commonly seen, leading to a three-year progression-free survival (PFS) of 12%, and a five-year overall survival of 19% [58]. Smaller studies of AR-positive SDC patients have reported somewhat less favorable outcomes, with an overall ADT response rate of 25%–50% [8,59]. Several case reports have shown a good effect of ADT alone in patients with AR-positive SDC or AC NOS, including stable disease for several months as well as cases of complete remission [43,60,61]. A few patients with SDC or AC NOS, who initially responded to a combination of bicalutamide and triptorelin but had a relapse, then showed a response to subsequent abiraterone, suggesting resistance mediated by the reactivation of AR signaling during ADT treatment [62,63]. ADT has also been combined with either definitive RT or palliative chemotherapy with robust responses in several single case reports of SGC [64,65].

Patients with AC NOS have been found to respond well to ADT, with partial or complete response in 10 of 11 reported cases, and a median PFS of 20 months. SDC patients appear to have a lower response rate, with partial or complete response in 11 of 26 (42%) reported cases and a median PFS of eight months (Table 2).

Of note, several dramatic responses to ADT in SGC patients were published only as case reports of extraordinary responders. A recent preliminary study including all SDC patients treated with ADT in the Netherlands showed somewhat more modest results, with partial response in four (13%) cases, stable disease in 10 (32%) cases, and progressive disease in 17 (55%) cases, and a median PFS of 3.8 months [45]. On the other hand, since the majority of SGCs are chemotherapy-resistant, the treatment options for patients with generalized disease are limited and AR is the most promising target for these patients with otherwise incurable disease. Several clinical trials are currently ongoing, investigating the efficacy of ADT in patients with recurrent/metastatic AR-positive SGC, using abiraterone, bicalutamide or enzalutamide in male and female patients (NCT02749903, NCT01969578, NCT02867852). In addition to providing valuable clinical response information, these trials will also collect tumor tissue for correlative research, facilitating further understanding of molecular determinants of response to ADT in AR-positive SGC.

## 6. Conclusions

AR is expressed in a majority of SDCs and in a minority of other SGCs such as AC NOS, and ADT has emerged as a promising therapy in patients with AR-positive SGC. Several potential mechanisms of resistance to ADT have been described, including the expression of AR splice variants and mutations in *FOXA1* and *FASN*. Ongoing and future clinical trials will likely shed light on the clinical benefit and limitations of ADT in AR-positive SGC.

## Figures and Tables

**Table 1:** Prevalence of positive AR immunoreactivity in different types of SGC.

Histology	AR Positivity ^1^	Reported Range ^2^	References
SDC	615/713 (86%)	43%–100%	[8,24,25,26,27,28,29,30,31,33,34,41,42,43,44,45]
AC NOS	11/43 (26%)	21%–33%	[28,33,34]
AcCC	6/40 (15%)	0%–31%	[28,32,34,35]
MEC	7/135 (5%)	0%–20%	[27,28,32,33,34,36]
ACC	7/145 (5%)	0%–20%	[28,32,33,34,36]
EMC	0/6 (0%)	N/A	[28]
MECA	0/7 (0%)	N/A	[28,33]
BCAC	2/2 (100%)	N/A	[32]
PLGA	1/2 (50%)	N/A	[28]

^1^ Number of AR-positive cases/total number of cases, in all studies combined; ^2^ Range of prevalence detected in the different studies. SDC, salivary duct carcinoma; AC NOS, adenocarcinoma not otherwise specified; AcCC, acinic cell carcinoma; MEC, mucoepodermoid carcinoma; ACC, adenoid cystic carcinoma; EMC, epithelial-myoepithelial carcinoma; MECA, myoepithelial carcinoma; BCAC, basal cell adenocarcinoma; PLGA, polymorphous low grade adenocarcinoma.

**Table 2:** Reported cases of ADT treatment in patients with AR-positive SGC.

Patient ID ^1^	Histology	Sex	Age ^2^	ADT Agents	Response	PFS (Months)	Ref.
1	AC NOS	m	73	Bicalutamide + triptorelin	CR	N.K.	[60]
2	AC NOS	m	72	Bicalutamide + triptorelin	CR	2	[58]
3	AC NOS	m	N.K.	Goserelin	PR	N.K.	[61]
4	AC NOS	m	59	Bicalutamide + triptorelin	PR	12	[63]
5	AC NOS	m	44	Bicalutamide + triptorelin	PR	25	[63]
6	AC NOS	m	67	Bicalutamide + triptorelin	PR	22	[58]
7	AC NOS	m	67	Bicalutamide + triptorelin	PR	22	[58]
8	AC NOS	m	46	Bicalutamide + triptorelin	PR	58	[58]
9	AC NOS	m	49	Bicalutamide + triptorelin	PR	7	[58]
10	AC NOS	m	62	Bicalutamide + triptorelin	PR	9	[58]
11	AC NOS	m	69	Bicalutamide + triptorelin	SD	20	[58]
12	Cyst AC	m	79	Bicalutamide + triptorelin	PR	14	[58]
13	Cyst AC	f	68	Triptorelin + cyproterone	PD	0	[58]
14	Poor diff.	m	54	Bicalutamide + triptorelin	PD	0	[58]
15	SDC	f	87	Bicalutamide + leuprolide ^3^	CR	24	[64]
16	SDC	m	44	Bicalutamide + triptorelin	CR	39	[58]
17	SDC	m	67	Bicalutamide + triptorelin	CR	11	[58]
18	SDC	m	66	Bicalutamide	PR	14	[43]
19	SDC	m	50	Bicalutamide	PR	8	[59]
20	SDC	f	83	Bicalutamide	PR	26	[59]
21	SDC	m	45	Goserelin	PR	4	[62]
22	SDC	m	45	Bicalutamide + goserelin	PR	10	[62]
23	SDC	m	45	Abiraterone + goserelin	PR	10	[62]
24	SDC	m	51	Bicalutamide + triptorelin	PR	6	[58]
25	SDC	m	67	Bicalutamide + triptorelin	PR	7	[58]
26	SDC	f	68	Bicalutamide + leuprolide	SD	17	[8]
27	SDC	m	57	Bicalutamide	SD	14	[59]
28	SDC	m	56	Bicalutamide + goserelin	SD	12	[59]
29	SDC	m	67	Bicalutamide + goserelin	SD	8	[59]
30	SDC	m	75	Bicalutamide + triptorelin	SD	8	[58]
31	SDC	m	54	Bicalutamide + triptorelin	SD	10	[58]
32	SDC	m	68	Bicalutamide + triptorelin	SD	23	[58]
33	SDC	f	48	Bicalutamide + leuprolide	PD	0	[8]
34	SDC	f	69	Bicalutamide + leuprolide	PD	0	[8]
35	SDC	m	77	Bicalutamide + leuprolide	PD	0	[8]
36	SDC	m	73	Bicalutamide + goserelin	PD	0	[59]
37	SDC	m	68	Bicalutamide + goserelin	PD	0	[59]
38	SDC	f	64	Bicalutamide	PD	0	[59]
39	SDC	m	39	Bicalutamide	PD	0	[59]
40	SDC	m	73	Bicalutamide	PD	0	[59]

^1^ Patients are sorted by tumor histology and then best response; ^2^ At start of ADT; ^3^ This patient received external beam radiotherapy together with ADT. ADT, androgen deprivation therapy; PFS, progression-free survival; Ref., reference; AC NOS, adenocarcinoma not otherwise specified; Cyst AC, cystadenocarcinoma; Poor diff., poorly differentiated; SDC, salivary duct carcinoma; m, male; f, female; N.K., not known; CR, complete response; PR, partial response; SD, stable disease; PD, progressive disease.
